# Inequalities in socioeconomic status and race and the odds of undergoing a mammogram in Brazil

**DOI:** 10.1186/s12939-016-0435-4

**Published:** 2016-09-15

**Authors:** Enirtes Caetano Prates Melo, Evangelina Xavier Gouveia de Oliveira, Dóra Chor, Marilia Sá Carvalho, Rejane Sobrino Pinheiro

**Affiliations:** 1 Department of Epidemiology, National School of Public Health, Oswaldo Cruz Foundation -DEMQS/ENSP, Rua Leopoldo Bulhões, 1480, room 806. Manguinhos, Rio de Janeiro, RJ 21041-210 Brazil; 2 Health Information and Networks Research Group, Oswaldo Cruz Foundation, Rio de Janeiro, RJ Brazil; 3 Scientific Computing Program, Oswaldo Cruz Foundation, Rio de Janeiro, RJ Brazil; 4 Institute for Studies in Collective Health, Federal University of Rio de Janeiro, Rio de Janeiro, RJ Brazil

**Keywords:** Mammogram, Health inequalities, Access to health services, Health equity, Information systems, Race, Brazil

## Abstract

**Background:**

Access to mammograms, in common with other diagnostic procedures, is strongly conditioned by socioeconomic disparities. Which aspects of inequality affect the odds of undergoing a mammogram, and whether they are the same in different localities, are relevant issues related to the success of health policies.

**Methods:**

This study analyzed data from the 2008 PNAD - Brazilian National Household Sample Survey (11.607 million women 40 years of age or older), on having had at least one mammogram over life for women 40 years of age or older in each of Brazil’s nine Metropolitan Regions (MR), according to socioeconomic position. The effects of income, schooling, health insurance and race in the different regions were investigated using multivariate logistical regression for each region individually, and for all MRs combined. The age-adjusted odds of a woman having had a mammogram according to race and stratified by two income strata (and two schooling strata) were also analyzed.

**Results:**

Having a higher income increases four to seven times a woman’s odds of having had at least one mammogram in all MRs except Curitiba. For schooling, the gradient, though less steep, is favorable to women with more years of study. Having health insurance increases two to three times the odds in all MRs. Multivariate analysis did not show differences due to race (except for the Fortaleza MR), but the stratified analysis by income and schooling shows effects of race in most MRs, with greater differences for women with higher socioeconomic status.

**Conclusions:**

This study confirms that income and schooling, as well as having health insurance, are still important determinants of inequality in health service use in Brazil. Additionally, race also contributes to the odds of having had a mammogram. The point is not to isolate the effect of each factor, but to evaluate how their interrelations may exacerbate differences, generating patterns of cumulative adversity, a theme that is still little explored in Brazil. This is much more important when we consider that race has only recently started be included in analyses of health outcomes in Brazil.

## Background

Screening procedures, such as mammograms, are essential for early detection of breast cancer, the most common type of cancer among women in Brazil and other countries. As with other diagnostic procedures, access to mammograms is strongly conditioned by socioeconomic position [1]. The difficulty in accessing screening is related to a lower socioeconomic status, increasing the odds of a late diagnosis, at a more advanced stage [2–4]. The persistence of population groups at risk for sub-utilization of mammograms may be considered an important health inequity (i.e. ethically unacceptable) affecting women and their families, as the procedure is available and positively impacts the disease, reducing mortality by up to 20 % [4].

Brazil is a country of continental dimensions, with around 200 million inhabitants, over 80 % of whom live in urban areas. The country’s 27 states are usually grouped into five regions: North, Northeast, Midwest, Southeast and South [5]. The last two are the richest and most developed, while the North and Northeast regions fall at the other end of the wealth and development spectrum. The smallest administrative divisions in the country are municipalities, encompassing both urban and rural areas [6]. Brazil is both highly urbanized and highly unequal. Whereas about 85 % of the 5563 municipalities have less than 20,000 inhabitants, nine Metropolitan Regions (MR) concentrate about 30 % of the population. These patterns of concentration are also manifest in terms of income, gross domestic product, and availability of services.

Since the creation of the Unified Health System (SUS) in 1988 by the Brazilian Federal Constitution, the public system is charged with ensuring full access to free, universal health care for all citizens. However, the resource distribution policies implemented since its inception were insufficient to reverse historically strong regional patterns of inequality, and recent studies have reported a marked inequality in the distribution of health care between regions [5].

In Brazil, a recent study showed a reduction in income and schooling inequalities in the mammogram coverage of the female population [6]. However, inequities in access to health services can still be observed both with regard to socioeconomic position and also among those living in different regions of the country. The study also found that living in a metropolitan region doubled the odds of undergoing the examination, when compared with living in an urban area. The study [7] did not find statistically significant differences for race regarding the odds of having had a mammogram. However, miscegenation is part of Brazilian history [8] and Brazil is a multiracial society in which race is a social construct that includes several dimensions of individuals’ lives and is a part of the social stratification that defines differences to access to goods and services. The scale for data gathering and analysis is not always that of determinants. It is therefore possible that analysis scale adopted by the study did not distinguish between the complex ways in which race/ethnicity and socioeconomic position combine, configuring different bases for exclusion and social stratification which affect the distribution patterns of health issues. At another level of determination, beyond individual characteristics, regional differences in health service use may be determined by the regions’ development level as well as by regional organization of services and health policies [9–11]. Certainly, in Metropolitan Regions, there is greater offer of services and accessibility problems have less to do with distance. Because they possess specific characteristics and concentrate an important portion of the population, metropolitan regions are a relevant scale for analyzing the determinants of differences in the odds of having a mammogram [4].

This study analyzed the odds of having had at least one mammogram over life for women 40 years of age or older in each of Brazil’s nine major Metropolitan Regions, according to socioeconomic position and race. Our aim was to explore the effect of income, education and race in different metropolitan settings, seeking to identify if there is a pattern of inequity and, if so, if this pattern varies according to Metropolitan Region.

## Methods

This is a cross-sectional study about socioeconomic determinants of access to mammograms in Brazil based on data from the Health Supplement of the 2008 National Household Sample Survey (PNAD) [12]. We analyzed having had at least one mammogram over life in a sample of 11.607 million women aged 40 years or older living in the Metropolitan Regions investigated in the PNAD. Metropolitan Regions are areas of occupation directly polarized by a metropolis which works as a center of command and coordination of an urban network. In Brazil, these regions have legal status. The nine Metropolitan Regions originally instituted in the 1970’s are the main urban areas in the country and are a representative stratum for IBGE’s household surveys, such as the PNAD. We chose to limit the analysis to Metropolitan Regions (MR), where geographic barriers to access are less important, but which still enable us to evaluate the effects of macro-regional differences on the odds of undergoing a mammogram.

We studied having had the examination according to race/skin color, as a representative of one of the axes of social stratification. “Race/skin color” is the terminology adopted by the Brazilian Institute of Geography and Statistics (IBGE), which is responsible for the Census and national surveys. The classification used in the Brazilian census mixes skin color and ethnicity. Since this a social construct, it is not a marker for genetic variation. Skin color refers to physical appearance, not origin, and is therefore a context-dependent attribute. The covariate race/skin color in PNAD is self-declared, with the following categories: White, Brown, Black, Yellow and Indigenous. In this classification, “Indigenous” may refer to physical characteristics, ancestry/ethnicity, or group identity. “Yellow” refers to Asian-Brazilians. The category “Brown” encompasses a broad spectrum of phenotypes and does not strictly refer to African ancestry.

The Brazilian Health Ministry guidelines for breast cancer screening recommend yearly clinical examinations of breasts for asymptomatic women starting at 40 years of age and biannual mammograms for women between the ages of 50 and 69 [13]. However, the Brazilian Society of Mastology recommends yearly examination starting at 40 years of age. Considering the elevated percentages of mammograms among women 40 years of age or older [14], the age ranges were established as 40–49 years, 50–69 years and 70 years or older.

Income and schooling were used as indicators of socioeconomic position. Income was measured as per capita monthly family income. The income of all family members, divided by the number of family members - a family measure attributed to an individual – is possibly a better expression of Socioeconomic position. This measure was stratified in six classes, based on the value of the 2008 legal minimum wage (MW) (R$415.00). Schooling was measured in six categories based on years of study.

We calculated the raw prevalences of having had a mammogram according to race/skin color, income and schooling.

**Fig. 1:**
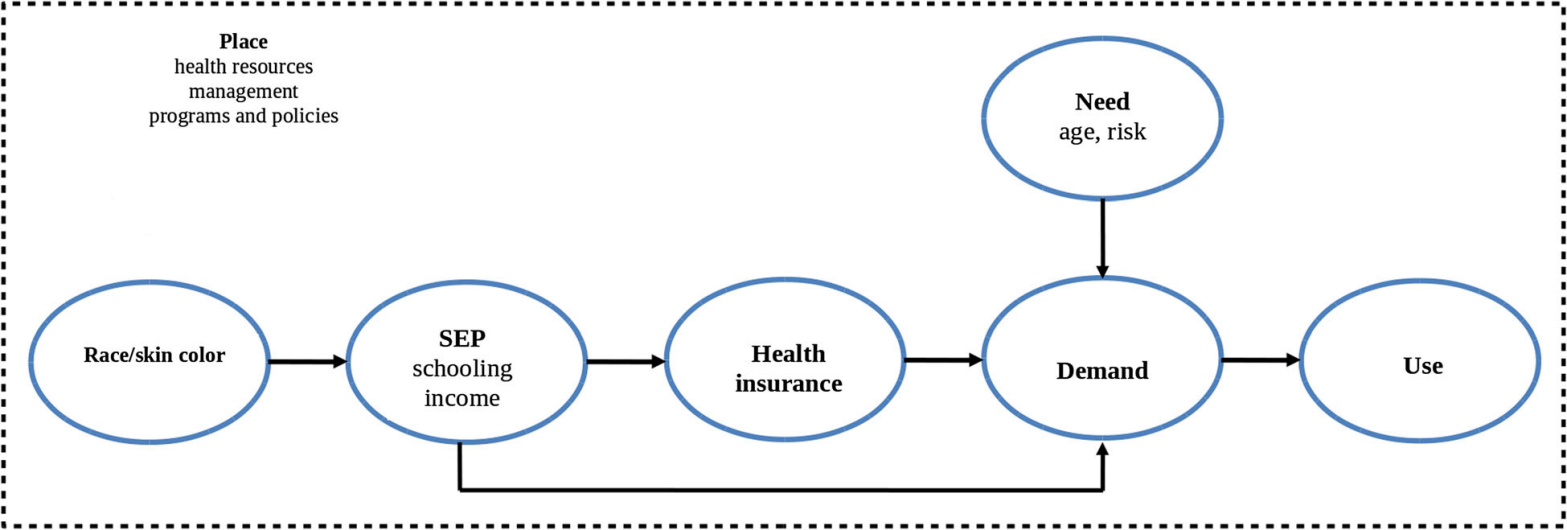
Conceptual framework of individual factors related to having had a mammogram

Several individual-level barriers are associated with screening adherence. For this study, the conceptual framework considered individual factors that influence having a mammogram and its timing over a person’s life (Fig. 1) [15–18]. Race/skin color was considered the most distal, with income, schooling and health insurance as intermediate covariates. Context may influence the way in which individual-level factors associate with the odds of having a mammogram.

The effects of race/skin color, income, schooling and health insurance were mutually adjusted and adjusted by age. The odds of ever having had a mammogram were estimated through multivariate logistical regression for all MRs combined and for each region individually. We estimated odds ratios (OR) and 95 % confidence intervals. The reference categories were 40–49 years, white, no schooling, income less than a quarter of the minimum wage (R$104.00) and not having health insurance. We also analyzed the age-adjusted odds of a woman having had a mammogram according to race/skin color and to two income strata as well as by two schooling strata. Stratification by per capita family income considered two groups, below and above ½ of the minimum wage (R$208.00). In the São Paulo and Curitiba MRs, however, the number of women in the lower stratum was insufficient for estimating the model. For these MRs, we used the minimum wage (R$ 415.00) as the cutoff point. For schooling, the cutoff point was 8 years.

We used the number of mammography units per million women aged 40 years or older to describe the provision of health service in each MR. This was based on data from the 2009 Survey of Medical-Sanitary Assistance (IBGE) and the 2009 population estimates (Projeto UNFPA/IBGE), available through the Information Technology Department of the Public Health Care System -SUS (DATASUS), at http://www.datasus.gov.br.

PNAD’s sample design is complex, using stratification, conglomeration, unequal probabilities of selection and adjustments of sample weights for calibration with known population totals. Thus, we had to correct the effects of the sample design [19]. Statistical analysis was carried out using the free software R (*survey* and SOAR library functions). We estimated the variance of strata composed of a single primary sample unit through the average of the remaining strata, using the option “*adjust*” from the *survey* library. Case selection for analysis was based on the corrected database.

## Results

**Fig. 2:**
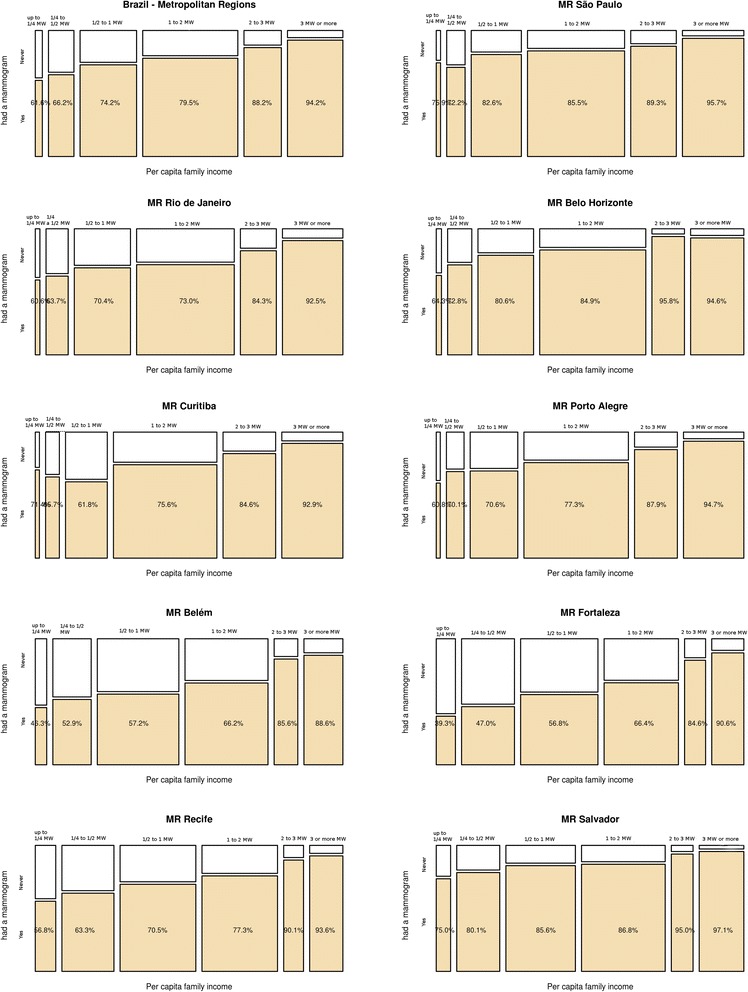
Mammogram raw prevalence according to per capita family income, by Metropolitan Region, Brazil, 2008

**Fig. 3:**
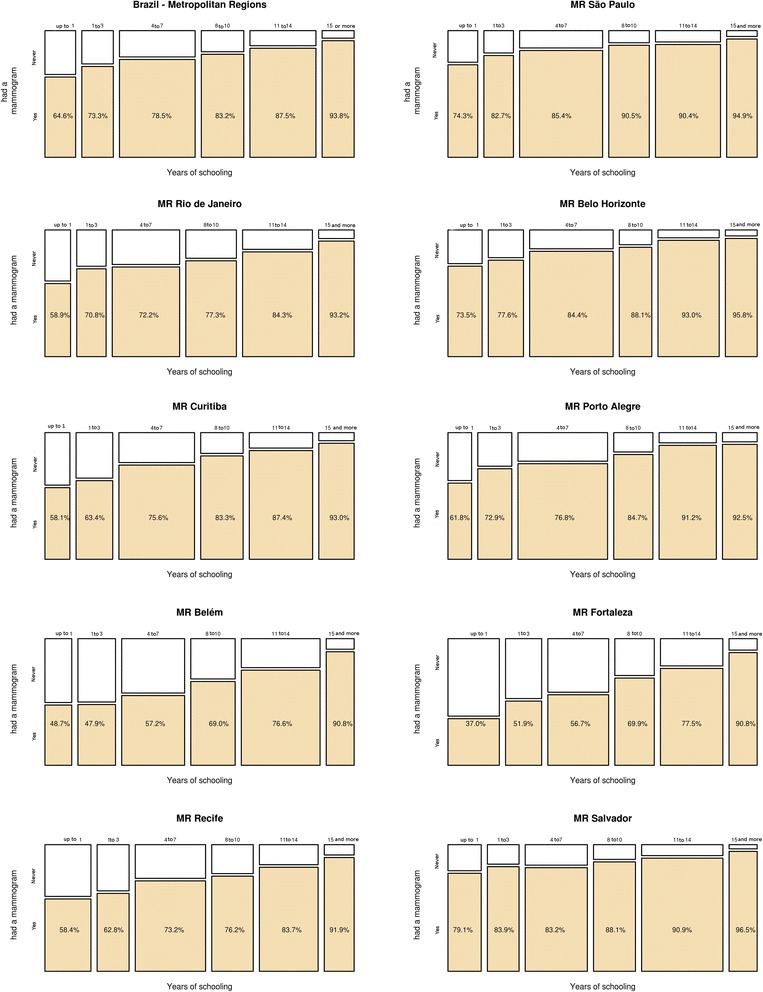
Mammogram raw prevalence according to schooling, by Metropolitan Region, Brazil, 2008

**Fig. 4:**
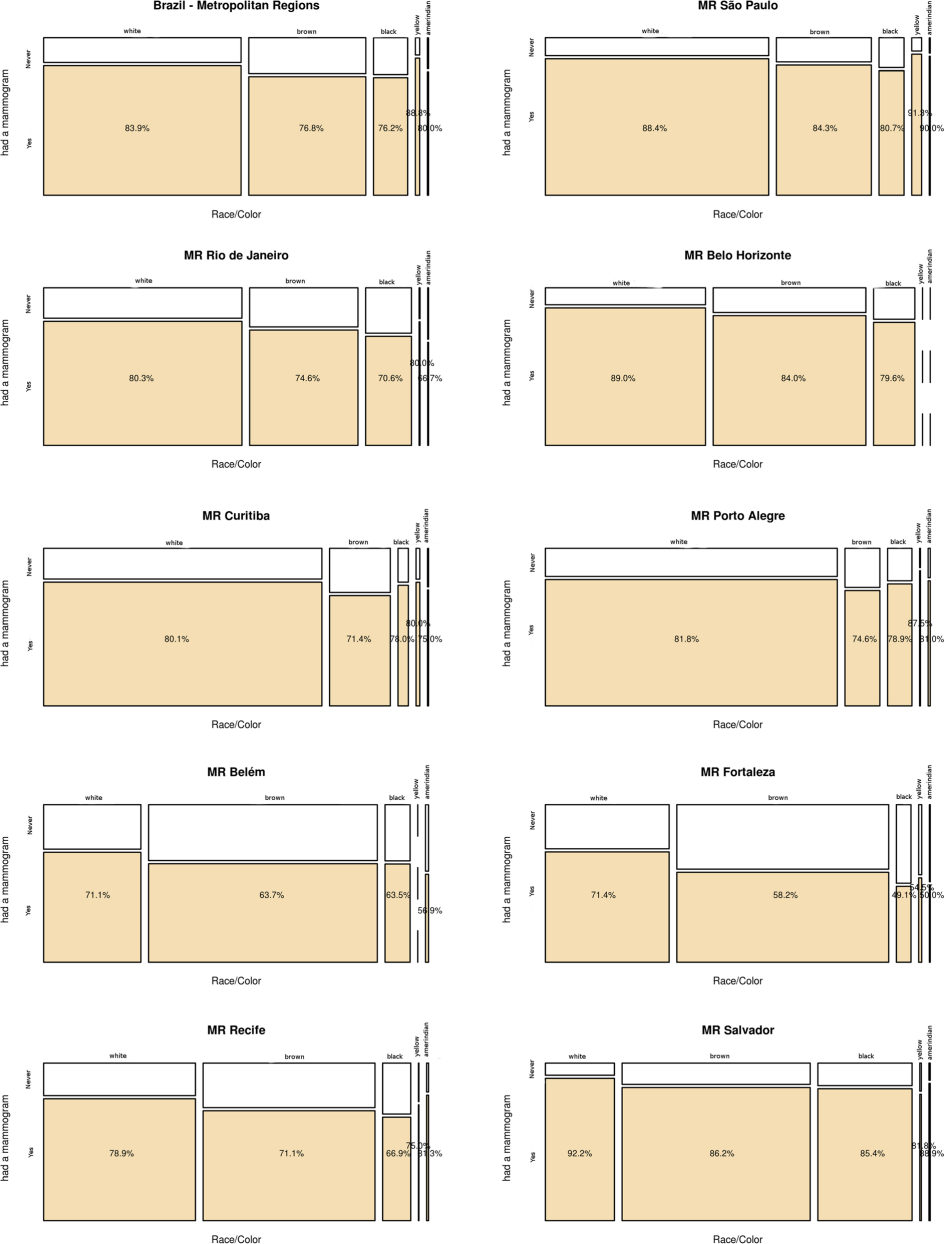
Mammogram raw prevalence according to race/skin color, by Metropolitan Region, Brazil, PNAD 2008

Figures 2, 3 and 4 show the raw prevalences of mammograms in the Metropolitan Regions, according to race/skin color, income and schooling.

**Fig. 5:**
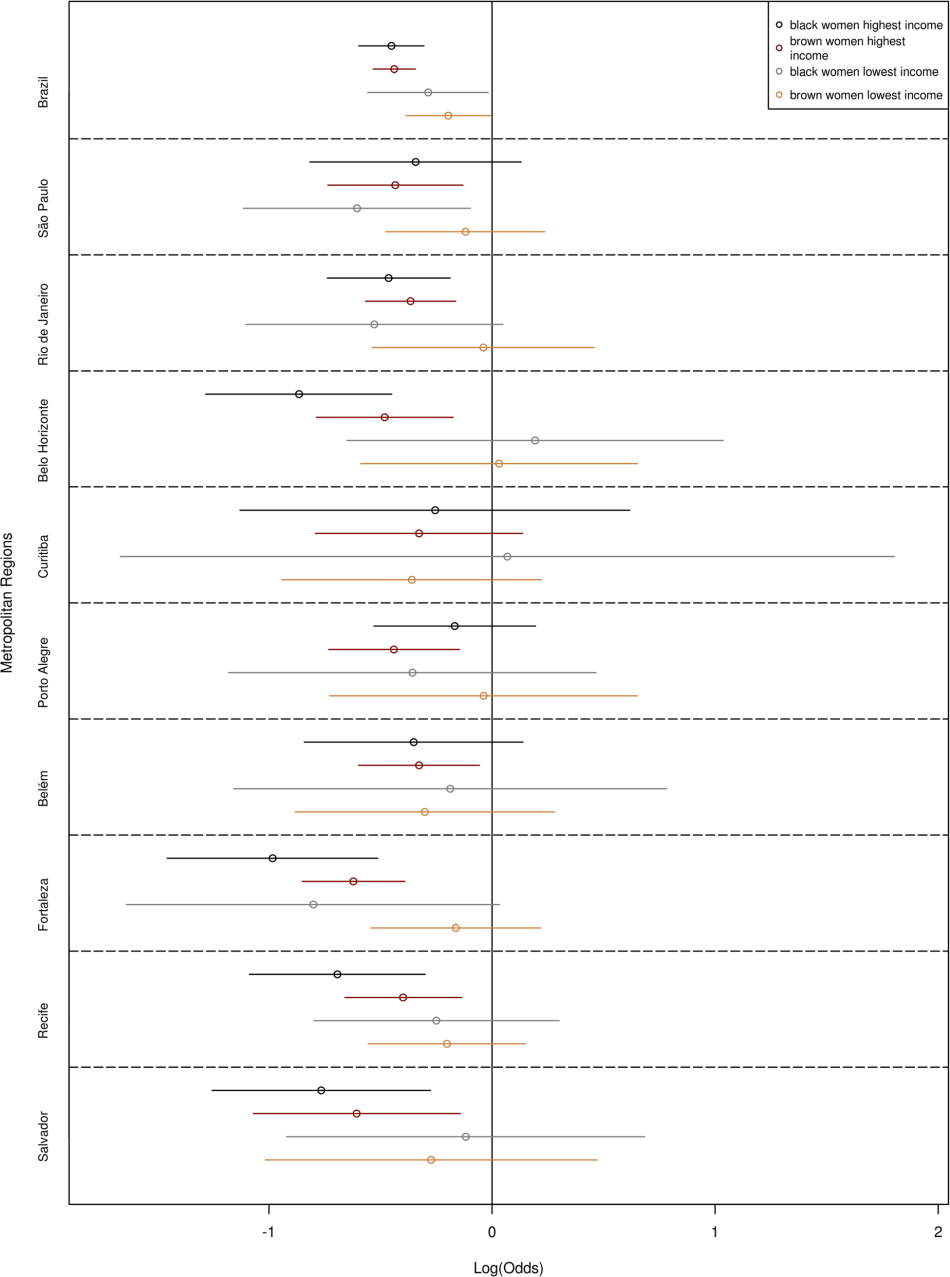
Effect of race/skin color, stratified by income, on mammogram use, by Metropolitan Region, Brazil, 2008

Family income is an important indicator of inequities in undergoing mammograms. Figure 2 shows the prevalences of having had at least one mammogram over life in the vertical axis and the per capita family income bands in the horizontal axis. The width of each band represents the number of women at each level of income in 2008. Within all MRs combined, there is a clear gradient of increasing mammogram prevalence following an increase in income, as shown by the fact that only 62 % of women with up to ¼ MW had had a mammogram; among those with between 1 and 2 MW (the largest category), 79.5 % had had a mammogram, and among those with the highest income, 94 % had had one.

For each of the MRs, the direct relationship between income and mammogram prevalence is repeated. Nonetheless, there are clear differences between them. In São Paulo, for example, 76 % and 96 % of women at the lowest and highest income bands, respectively, had had a mammogram. At the other extreme, in Fortaleza, the prevalence ranged from 39 % to 91 %. Therefore, having a lower income is associated with lower mammogram usage in Fortaleza, Belém (46 % and 89 %) and Recife (57 % and 94 %) as compared to São Paulo, Curitiba (71 % and 93 %) and Salvador (75 % and 97 %), the latter of which differs from the pattern of MRs located in the Northeastern region of the country.

We found the same kind of relationship between schooling and proportion of women who had had a mammogram (Fig. 3). For all MRs combined, 65 % of women with less than one year of schooling had had a mammogram, against 94 % of those with 15 years of schooling or more. The differences in Fortaleza (37 % and 91 %) and Belém (49 % and 91 %) were greater than those in São Paulo (74 % and 95 %) and Belo Horizonte (74 % and 96 %).

Women who self-identify as brown, black or indigenous are at a disadvantage regarding those who identify as white or yellow. As with the other indicators, there are large differences among the MRs. There are regional patterns that oppose higher prevalences of having had the examination in the MRs located in the South and Southeast regions, to lower prevalences in those located in the North and Northeast regions, once again with the exception of Salvador, with prevalences similar to those of São Paulo. In Fortaleza, mammogram prevalences were 49 % (black), 50 % (indigenous), 58 % (brown), and 71 % (white); in São Paulo, they were 81 % (black), 84 % (brown), 91 % (yellow) and 88 % (white) (Fig. 4).

Per capita family income, adjusted by age, race/skin color, health insurance and schooling had an independent effect and direct correlation: the higher the income, the higher the odds of having had a mammogram in the MRs (Table 1). At the highest income bracket (3 MW or more), the odds of a woman having had a mammogram were four times higher (OR = 4.10; 95 % CI: 3.20–5.24) than those situated at the lowest income bracket (less than ¼ MW) for all MRs combined. We found important variations among the MRs, with effects at ½ MW, 1 MW and 2 MW. Unlike the other MRs, income did not have a significant effect in Curitiba. The biggest differences, when comparing the highest and lowest income brackets, were found in Porto Alegre (OR = 5.06; 95 % CI: 2.53–10.15). Schooling also had a direct correlation and independent effect on the odds of having a mammogram in all MRs combined, though with a weaker association than income (Table 1). This result suggests that inequalities in schooling have a smaller impact on having the examination. The odds for women with 15 years of schooling or more was two times higher than the odds for those with less than one year of schooling (OR = 2.33; 95 % CI: 1.85–2.93). The difference between the lowest and the highest schooling strata ranged from almost two to three times higher, in almost all MR. Fortaleza showed the biggest difference (OR = 5.12; CI: 3.00–8.75). In Curitiba, where income did not have an independent effect, schooling significantly influenced having had a mammogram starting at the “4 to 7 years of schooling” category (OR = 1.85;(OR = 2.03; 95 % CI: 1.18–2.88), with a well-defined rising gradient. In Salvador, on the other hand, there was a marked income difference, and schooling did not have a significant effect.

**Table 1:** Multivariate logistical regressions of factors related to having had a mammogram in women 40 years of age or older, Brazil, Metropolitan Regions, 2008

Variables	Metropolitan Regions combined	MR São Paulo	MR Rio de Janeiro	MR Belo Horizonte	MR Curitiba	MR Porto Alegre	MR Belém	MR Fortaleza	MR Recife	MR Salvador
Age range										
40–49 years	1.00	1.00	1.00	1.00	1.00	1.00	1.00	1.00	1.00	1.00
50 to 69 years	**1.48**	**1.54**	**1.36**	**1.73**	**1.56**	**1.65**	**1.40**	**1.63**	**1.81**	**1.70**
70 years or more	**0.62**	**0.51**	**0.65**	0.69	0.79	**0.72**	0.70	0.84	0.80	0.67
Race/skin color										
white	1.00	1.00	1.00	1.00	1.00	1.00	1.00	1.00	1.00	1.00
brown	**0.91**	0.95	1.01	0.90	0.95	0.91	1.00	0.84	0.87	0.82
black	0.91	0.76	0.88	0.77	1.14	0.99	1.03	**0.57**	0.82	0.81
yellow	1.13	1.05	0.64		0.77	0.58		0.63	0.75	0.43
indigenous	1.04	1.38	0.49		1.08	0.90	0.64	0.74	1.44	1.04
Years of schooling completed										
less than 1	1.00	1.00	1.00	1.00	1.00	1.00	1.00	1.00	1.00	1.00
1 to 3	**1.34**	**1.47**	**1.52**	1.09	1.12	**1.46**	0.81	**1.72**	1.17	1.28
4 to 7	**1.51**	**1.43**	**1.38**	**1.51**	**1.85**	**1.59**	1.19	**2.14**	**1.79**	1.10
8 to 10	**1.86**	**2.15**	**1.66**	**1.78**	**2.74**	**2.32**	**1.74**	**3.21**	**1.89**	1.55
11 to 14	**1.97**	**1.56**	**2.05**	**2.42**	**3.02**	**3.15**	**2.11**	**3.55**	**2.06**	1.44
15 and more	**2.33**	**1.85**	**2.61**	**3.11**	**3.45**	**2.41**	**3.22**	**5.12**	**2.34**	1.67
Per capita family income										
up to 1⁄4 MW	1.00	1.00	1.00	1.00	1.00	1.00	1.00	1.00	1.00	1.00
1⁄4 to 1/2 MW	1.19	0.82	1.09	1.40	0.68	1.54	1.19	1.24	1.17	1.32
1⁄2 to 1 MW	**1.56**	1.31	1.37	**2.07**	0.46	1.35	1.31	**1.51**	**1.45**	**1.92**
1 to 2 MW	**1.91**	1.53	1.46	**2.50**	0.75	**1.77**	**1.68**	**1.97**	**1.78**	**1.75**
2 to 3 MW	**2.76**	**1.72**	**2.28**	**6.76**	0.90	**3.04**	**3.26**	**3.08**	**3.16**	**3.64**
3 MW or more	**4.10**	**3.63**	**3.12**	**3.41**	1.46	**5.06**	**2.69**	**3.56**	**2.89**	**3.88**
Health insurance										
No	1.00	1.00	1.00	1.00	1.00	1.00	1.00	1.00	1.00	1.00
Yes	**2.48**	**2.11**	**2.70**	**2.26**	**2.72**	**2.00**	**2.67**	**2.64**	**2.99**	**3.07**

Highlighted values, significant at 5 %

Having health insurance doubles the odds of having a mammogram in all MRs combined. The odds varied between 2.00 (95 % CI: 1.56–2.57) in Porto Alegre and 3.07 (95 % CI: 2.03–4.63) in Salvador. Generally speaking, the highest values were observed in the MRs located in the country’s poorest regions.

Stratifying for income (Table 2 and Fig. 5), among women with a per capita family income of less than ½ MW (or 1 MW in São Paulo and Curitiba, Table 3), the odds of black and brown women having had a mammogram were, respectively, 25 % (OR = 0.75; 95 % CI: 0.57–0.98) and 18 % (OR = 0.82; 95 % CI: 0.68–0.99) lower than those of white women. Among women with the lowest income, however, there were no significant differences between black, brown and white women, and this holds for every MR.

**Table 2:** Multivariate logistic regression, race/skin color stratified by per capita family income, having had a mammogram for women 40 years of age or older (OR), Brazil, Metropolitan Regions, 2008

Metropolitan Regions	Age group		Race/skin color	
	50 to 69 years	70 years or more	brown	black
Per capita family income: up to 1⁄2 MW				
Metropolitan Regions combined^a^	**1.37**	**0.61**	**0.82**	**0.75**
MR Rio de Janeiro	1.00	0.80	0.96	0.59
MR Belo Horizonte	1.76	1.04	1.03	1.21
MR Porto Alegre	1.65	0.52	0.96	0.70
MR Belém	1.22	0.40	0.74	0.83
MR Fortaleza	**1.46**	**0.44**	0.85	0.45
MR Recife	**1.57**	0.87	0.82	0.78
MR Salvador	1.35	1.01	0.76	0.89
Per capita family income: 1⁄2 MW or more				
Metropolitan Regions combined^a^	**1.32**	**0.52**	**0.65**	**0.64**
MR Rio de Janeiro	**1.31**	**0.58**	**0.70**	**0.63**
MR Belo Horizonte	1.35	**0.53**	**0.62**	**0.42**
MR Porto Alegre	**1.45**	**0.57**	**0.64**	0.85
MR Belém	1.14	**0.54**	**0.72**	0.70
MR Fortaleza	1.22	**0.55**	**0.54**	**0.37**
MR Recife	**1.68**	**0.57**	**0.67**	**0.50**
MR Salvador	**1.76**	**0.61**	**0.55**	**0.47**

^a^Including the São Paulo and Curitiba Metropolitan Regions, with a cutoff value of 1 MW

Highlighted values, significant at 5 %

**Table 3:** Multivariate logistic regression, race/skin color stratified by per capita family income, having had a mammogram for women 40 years of age or older (OR), São Paulo and Curitiba Metropolitan Regions, 2008

Metropolitan Regions	Age range		Race/skin color	
	50 to 69 years	70 years or more	brown	black
Per capita family income: up to 1 MW				
MR São Paulo	**1.54**	**0.52**	0.89	**0.55**
MR Curitiba	0.99	0.64	0.70	1.07
Per capita family income: 1 MW or more				
MR São Paulo	1.20	**0.36**	**0.65**	0.71
MR Curitiba	1.23	**0.51**	0.72	0.78

Highlighted values, significant at 5 %

Among women with a per capita family income equal to or higher than ½ MW (or 1 MW in São Paulo and Curitiba), the odds of having had a mammogram were even lower for brown and black women, when compared to white women. For all MRs combined, they were around 35 % lower for brown and black women. In Belo Horizonte, Salvador and Recife, the odds were around 50 % and 40 % lower for black and brown women, respectively. In Fortaleza, the odds were 63 % lower for black women (OR = 0.37; 95 % CI: 0.23–0.60) and 47 % lower for brown women (OR = 0.54; 95 % CI: 0.43–0.68).

**Fig. 6:**
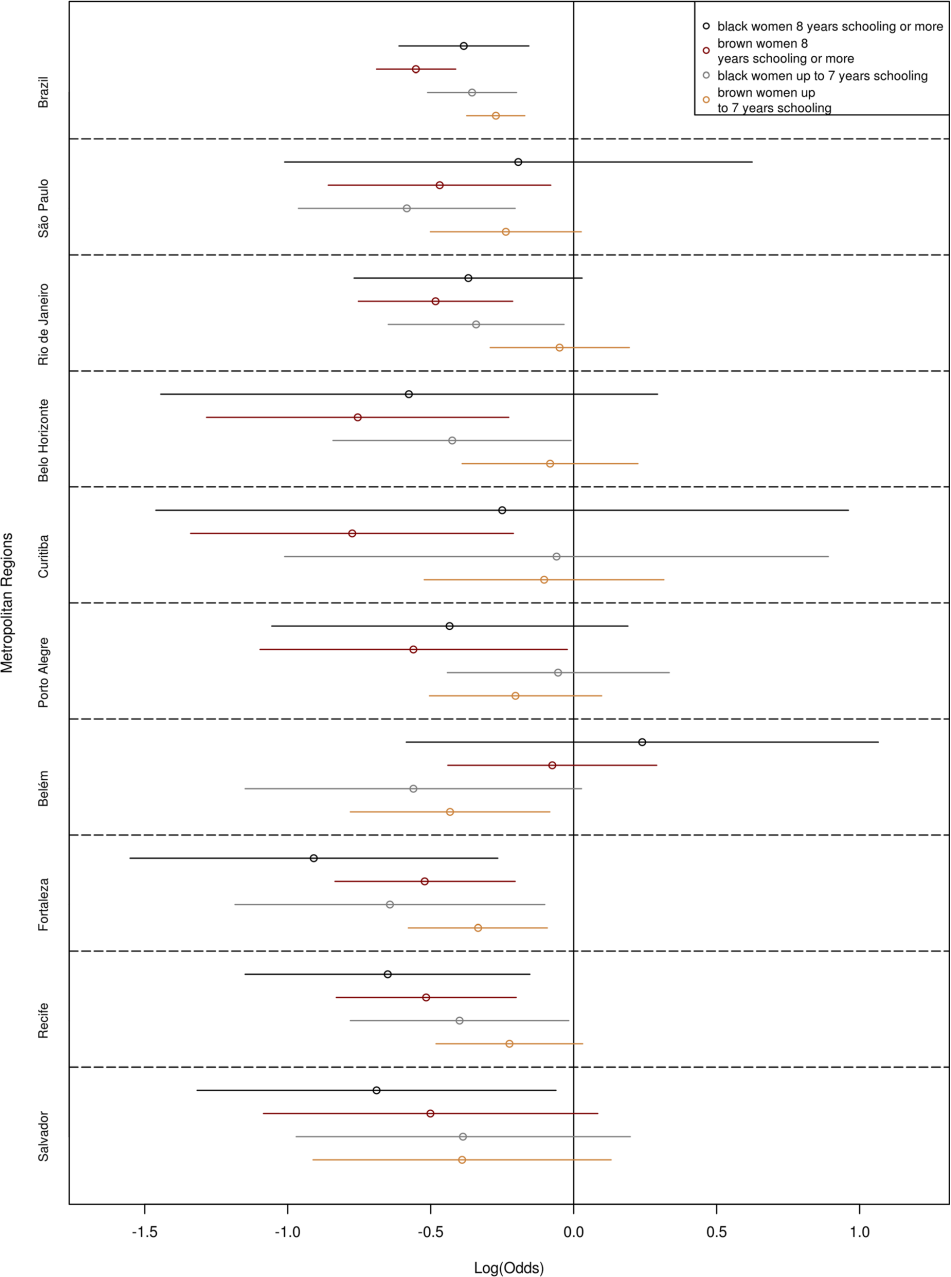
Effect of race/skin color, stratified by schooling, on mammogram use, by Metropolitan Region, Brazil, 2008

We also found that black and brown women were less likely to have had a mammogram when considering the schooling strata, but there were differences among the MRs. For women with up to seven years of schooling, the odds of having had a mammogram for black and brown women were, respectively, 30 % (OR = 0.70; 95 % CI: 0.60–0.82) and 24 % (OR = 0.76; 95 % CI: 0.69–0.84) lower than those of white women in all MRs combined. In comparison to white women, the pattern for MRs is that there is a difference for black women, but not for brown women. For example, in São Paulo, black women have nearly half the odds of white women (OR = 0.56; 95 % CI: 0.38–0.82), but there are no significant differences between brown and white women (OR = 0.79; 95 % CI: 0.61–1.03). The MRs that do not follow this pattern are Fortaleza, where black women (OR = 0.53; 95 % CI: 0.31–0.90) and brown women (OR = 0.72; 95 % CI: 0.56–0.91) have lower odds of having had a mammogram; Salvador, Curitiba and Porto Alegre, where there are no statistically significant differences; and Belém, where there are differences only between brown and white women (Table 4 and Fig. 6).

**Table 4:** Multivariate logistic regression, race/skin color stratified by schooling, having had a mammogram for women 40 years of age or older (OR), Brazil, Metropolitan Regions, 2008

Metropolitan Regions	Age range		Race/skin color	
	50 to 69 years	70 years or more	brown	black
Years of schooling: up to 7				
Metropolitan Regions combined	**1.56**	**0.76**	**0.76**	**0.70**
MR São Paulo	**1.64**	**0.55**	0.79	**0.56**
MR Rio de Janeiro	**1.34**	0.78	0.95	**0.71**
MR Belo Horizonte	**1.91**	1.02	0.92	**0.65**
MR Curitiba	1.23	0.75	0.90	0.94
MR Porto Alegre	**1.76**	0.83	0.82	0.95
MR Belém	1.23	0.87	0.65	0.57
MR Fortaleza	**1.56**	0.97	0.72	**0.53**
MR Recife	**1.77**	0.92	0.80	**0.67**
MR Salvador	**1.69**	0.95	0.68	0.68
Years of schooling: 8 or more				
Metropolitan Regions combined	**1.79**	0.95	**0.58**	**0.68**
MR São Paulo	**1.76**	1.17	**0.63**	0.82
MR Rio de Janeiro	**1.75**	0.96	**0.62**	0.69
MR Belo Horizonte	**1.83**	0.55	**0.47**	0.56
MR Curitiba	**2.46**	1.59	**0.46**	0.78
MR Porto Alegre	**1.90**	1.16	**0.57**	0.65
MR Belém	**1.87**	0.90	0.93	1.27
MR Fortaleza	**1.97**	1.24	**0.59**	**0.40**
MR Recife	**2.40**	0.98	**0.60**	**0.52**
MR Salvador	**2.38**	1.04	0.61	**0.50**

Highlighted values, significant at 5 %

Among women with 8 years of schooling or more, the odds of having had a mammogram for black and brown women were, respectively, 32 % (OR = 0.68; 95 % CI: 0.54–0.86) and 42 % (OR = 0.58; 95 % CI: 0.50–0.66) lower than those of white women in all MRs combined. This pattern is also different when we look at each MR separately. As a rule, there are significant differences between brown and white women, but not between black and white women, especially in Belo Horizonte and Curitiba, where brown women have half the odds of having had a mammogram as those of white women. The exceptions are Fortaleza and Recife, where, despite having 8 years of schooling or more, both groups differ in regard to white women. The odds of black women having had a mammogram are 60 % and 48 % lower in these MRs, respectively, and the odds of brown women in both MRs are 40 % lower. In Salvador, however, only black women have significantly lower odds (OR = 0.50; 95 % CI: 0.27–0.94) of having had a mammogram.

Table 5 shows data on mammograms in all nine MRs. The number of mammograms per million women aged 40 years or older is twice as high in MR with the highest ratio (Salvador, 183.6) as the MR with the lowest ratio (Recife, 98.6), both located in the Northeast Region, which has some of the worst standards of living in the country. The variation we found does not seem to be directly associated with the variation in mammogram prevalence, nor with the patterns of schooling, income and race/color inequality we identified.

**Table 5:** Distribution of mammography units in the nine Metropolitan Regions, in non-metropolitan regions and in Brazil, 2009

Region	Total number of mammography units	Number of women aged 40 years or older	Mammography units per million women
Brazil	3,089	31,721,764	97.4
Non-metropolitan	1,539	21,346,285	72.1
MR combined	1,550	10,375,479	149.4
RM Belém	34	308,820	110.1
RM Fortaleza	62	539,588	114.9
RM Recife	65	659,013	98.6
RM Salvador	110	598,978	183.6
RM Belo Horizonte	167	972,151	171.8
RM Rio de Janeiro	404	2.422,282	166.8
RM São Paulo	530	3.526,152	150.3
RM Curitiba	67	549,707	121.9
RM Porto Alegre	111	798,788	139.0

Source: IBGE

## Discussion

In spite of the reduction in levels of inequalities in Brazil over the past few decades [20], marked social and regional inequities still persist regarding the odds of undergoing a mammogram. Screening prevalence is higher for women with higher income, higher schooling levels, who are white or who live in areas with a higher socioeconomic profile, in accordance with the literature [11, 21, 22]. Differences among the MRs confirm the existence of “several Brazils” and different aspects of socioeconomic position (SEP) seem to condition mammogram use in each context. These aspects reflect more than just individual characteristics or the unbalanced spatial distribution of population and health care services. They also point to inequalities in the attributes of localities and in the use and quality of health care services, which may be modified through social policies, including health policy [22–24].

PNAD estimated that 4.7 million women aged between 50 and 69 years (28.9 % of the target population) have never had a mammogram. Women in this age group had higher odds of having a mammogram, when compared with women under 50. For women aged 70 years or older, the odds were lower. However, it must be noted that the effect of age measured in our analysis may be an effect of changing participation rates over time rather than an effect of age as such.

Unlike countries that adopted screening programs for breast cancer [25–29], Brazil is considered to have an “opportunistic” offer of screening. Since 2004, the model adopted in Brazil [30] defines the target population (women between the ages of 50 and 69) and the biannual periodicity of mammograms. However, there is no registry of the entire target population, women are not summoned to have an exam, and monitoring indicators are not systematically followed.

In Brazil, there was a significant expansion in the coverage of screening mammograms in the target age group. However, approximately 40 % of screening mammograms [14] carried out in the public health care system refer to women aged between 40 and 49 years. We must consider the controversial evidence [25, 31–33] concerning the efficacy of mammogram screening on the relative reduction of mortality due to breast cancer among women under the age of 50. Given the uncertainty regarding benefits, the high proportion of examinations carried out on women outside the target age group produces unnecessary spending for the health care system and potential harms from overdiagnosis and overtreatment resulting from screening.

The evaluation of the specific effect of recent changes to the various analytical dimensions on mammogram prevalence in the different age groups usually considers having had a mammogram over the past two years or over the past year to have a higher sensitivity, and evaluations of screening program’s impact have also investigated having had repeated mammograms within specific periods [34] . However, there is also evidence that having ever had a mammogram represents minimum access and this is the marker we selected [35].

Schooling, income and race/skin color are widely known to influence access to services, in Brazil as in other countries [7, 11, 12, 24]. Results differ according to the indicators considered, and this is one of the contributions of this study. In some Metropolitan Regions, mammogram prevalence is highly affected by per capita family income. In others, differences in mammogram prevalence are higher according to other dimensions of SEP, such as schooling. In each Metropolitan Region, barriers to mammograms may be different.

Income is the SEP indicator that is most directly related to obtaining material resources. Income has a dose-response effect on health [36, 37]. It influences both individuals’ ability to obtain a mammogram, through their ability to pay for it with their own resources, and their ability to travel and manage their time in order to undergo the examination. Income can vary over the life course and has a cumulative effect. It enables access not only to obtaining a procedure, but also influences the choice for the best procedure available. It is also the SEP indicator that may be most quickly altered through compensatory policies.

Schooling, in addition to conditioning occupation and income, acts at the level of information and other non-material resources associated with promoting healthy behaviors and seeking preventive care. It makes individuals more susceptible to messages on health education and qualifies communication, with direct effects on access to the appropriate health services.

Access is one of the determinants of health service use [38]. In Curitiba, the absence of an effect for income may be related to greater access to health services, both in terms of getting examination referrals as well as actually getting examinations performed. Barriers to accessing these services may be explained by income differences in the rest of the Metropolitan Regions, especially in São Paulo and Rio de Janeiro, favorable to women with at least twice the minimum wage, and Porto Alegre and Belém, favorable to women with at least minimum wage. For the other Metropolitan Regions, we observed a positive gradient starting at half the minimum wage. In these MR, small increases in income correspond to greater use. On the other hand, in Curitiba, we observed a difference in having had the examination according to schooling, which reflects differences in health behaviors and perception, with effects on seeking preventive services.

Income also influences access to material resources for maintaining health at the contextual level (household and neighborhood). In this study, we analyzed the effect of per capita family income, as sharing financial resources within a household may broaden or reduce the potential of an individual’s income. The neighborhood’s economic composition may act both through the availability of healthy living resources and through social networks, affecting the individual’s socioeconomic position. Each level contributes independently and is associated with the distribution of different types of exposures and health outcomes. Additionally, markers of socioeconomic position affect health indirectly. As an example, income influences health through its direct effect on obtaining material resources, which, in turn, influence more proximal factors in the causal chain, such as health behaviors, which are highly influenced by levels of schooling. Schooling also intensifies an individual’s capacity to find better occupation in the job market. Schooling captures the socioeconomic position an individual receives from their family, is a strong predictor of occupation and income [37] and makes individuals more receptive to health education messages, with direct effects on access to the appropriate health services.

In the analysis according to two income and schooling strata there is still some heterogeneity within groups. Thus, analyzing the odds of having had a mammogram in the income and schooling strata allows us to observe the effect of race/skin color, instead of eliminating it.

Because different SEP and race/skin color indicators do not capture the same phenomena, they must be investigated separately [9–11]. In fact, results suggest that race/skin color may be one of the barriers to undergoing mammograms, since residual differences are observed even after controlling for SEP, especially at the highest levels of income in the Metropolitan Regions. For the poorer segments, on the other hand, its influence seems to be weaker.

The conflation of socioeconomic inequalities and racial miscegenation leads to a very particular scenario in Brazil, which deserves to be studied. In this context, it is necesssary to identify the role of the social determinants of health (education, family income, social class), the effects of health inequalities and the interrelationships between socioeconomic and racial inequalities [39, 40]. Beyond the patterns of inequality in the country, we have to understand the role of cumulative adversity, a theme that is still little explored in Brazil. Differences in use between different race/skin color groups may occur due to the complexity of relationships between different groups and SEP. The effect may emerge from multiple mechanisms that affect health service use [40]. It should also be noted that these mechanisms act since birth in determining the individual’s socioeconomic position.

Though studies on this topic are rare in Brazil, the international literature shows that black women report lower service use and higher discrimination than other groups, affecting examination and service referrals as well as perception and behavior in seeking and using health care [37, 40–42]. In our study, too, considering the combined Metropolitan Regions, black and brown women had lower odds of having had a mammogram. Different proportions of black and brown population in each Metropolitan Region may be part of the explanation for differences in racial self-classification, as well as in discrimination in health services.

Additionally, other studies have shown an association between the region’s socioeconomic level and adherence to screening [43, 44] even when considering individual factors to adjust models. The social gradient penalizes the most disadvantaged groups [18]. Neighborhoods with lower socioeconomic levels have more limited support, whether in terms of service availability or quality, or in terms of social resources (such as social capital and cohesion), which are also associated with adherence to screening programs. Reducing inequalities in access to care and the availability of diagnostic and treatment methods may reduce breast cancer mortality [38, 45, 46].

Even though offer should be determined by health needs, some particular distortions related to the care model can be due to poor access. Just as offer may induce demand, in Brazil and elsewhere, the provision of health services in general is not distributed according to health needs, but follows the “Inverse Care Law” [47]. The provision of equipment and human resources does not seem to be a determining factor in explaining the variations in the chance of obtaining mammography in the context of the metropolitan regions. In Brazil, the number of mammography units nearly doubled between 2002 and 2009. About half of the country’s mammography units is located in the nine metropolitan regions and the number of units per million women in those areas is similar to that found in developed countries [48].

Since the information on having had a mammogram is self reported, it is possible there may have been a bias in the answers, due to different reasons, leading to an overestimation that may be different among the age and social groups.

Although this was not the focus of the study, data on mammogram prevalence for different age groups, in a specific period, would enable researchers to evaluate the screening program’s impact within specific periods [10]. It is worth noting that only 13.6 % of all women had a mammogram more than three years before the survey.

Given that this is a sectional study, screening prevalence will be influenced by survival determinants. The unequal probability of death in the exposure groups may generate different odds. Since it is expected that individuals with worse socioeconomic conditions have lower survival rates, individuals with lower income and schooling tend to be under-represented among survivors.

A potential source of error is related to the use of a respondent for the interview other than the woman herself. Due to logistical factors, PNAD does not include a second visit by the interviewer. For this reason, data on an absent inhabitant my be provided by those present at the household at the time of the interview.

At a time when the periodicity of screening by mammogram is under discussion in Brazil, and there are changes proposed to the national policy, we stress that this study focused on the analysis of access as reflected in ever having had a mammogram. Therefore, its conclusions would not be affected by changes seeking to reduce overuse.

This study confirms that income and schooling are still important determinants of inequities in health service use in Brazil. Additionally, we showed that race/skin color also contributes to the odds of having had a mammogram. We did not intend to isolate the effect of each factor, but to evaluate how their interrelations may exacerbate differences. This is even more important when we consider that race/skin color has only recently started to be included in analyses of health outcomes in Brazil.

## Conclusions

In Brazil, the control of breast cancer is a Ministry of Health defined priority. However, this objective is affected by inequalities in the use of services reflecting the strong social gradient unfavorable to persons in disadvantaged groups, in spite of recent reductions in the inequality burden. The structure of the health services network and the model adopted for local care have a key role in this setting. Organized screening programs produce equity in use through active surveillance, ensuring identification of the target population. In the Brazilian health system, the Family Health Strategy is responsible for the expansion, qualification and consolidation of primary care. This would be an appropriate setting for the necessary involvement of the public sector in the provision of examinations, tracking women with abnormal screening tests and regulation of the health care network. In this regard, the strengthening of SUS financing and redistributive policies should focus on profiling each population group according to their needs.

## Abbreviations

DATASUS: Information technology department of the public health care system -sus
IBGE: Brazilian institute of geography and statistics
MR: Metropolitan regions
MW: Minimum wage
PNAD: National household sample survey
SEP: Socioeconomic position
SUS: Unified health system
